# Presumed tuberculosis spondylitis with sternal involvement

**DOI:** 10.1016/j.radcr.2024.10.025

**Published:** 2024-11-15

**Authors:** Danielle Rossell, William Kim, Wayne Manness, Nitin Das Kunnathu Puthanveedu

**Affiliations:** a College of Medicine Rockford, University of Illinois, 1601 Parkview Ave., Rockford, IL 61107 USA; b College of Medicine Peoria, University of Illinois, 1 Illini Dr, Peoria IL 61605 USA; c Department of Neuroradiology, Central Illinois Radiological Associates, 111 Oakwood Road, East Peoria IL 61611, USA; d Department of Infectious Disease, University of Illinois College of Medicine Peoria, 1 Illini Dr, Peoria IL 61605, USA

**Keywords:** Tuberculosis, Spondylodiscitis, Sternum, Osteomyelitis, Pott's disease, Magnetic resonance imaging

## Abstract

Tuberculosis Spondylitis, also known as Pott's Disease, is an extrapulmonary form of tuberculosis (TB) that affects the spine. Sternal involvement is rare and accounts for only 0.3 % of cases. Its presentation is usually insidious in onset with many patients having little to no symptoms. Additionally, microbiological and histological results are inconsistent due to paucibacillary load from vertebral biopsies making definitive diagnosis challenging. Due to difficulty obtaining a diagnostic sample, a significant amount of TB spondylitis cases are presumed TB cases from improving clinical symptoms after empiric treatment as was our case. The purpose of this case report is to emphasize the diagnostic challenges of TB as well as present imaging findings over the course of two years with progressive involvement of the spine and sternum. Thus, here we report a case of presumed spinal tuberculosis affecting not only multiple levels of the spinal column but also the sternum in an immunocompetent United States born citizen with an indolent clinical course.

## Introduction

Tuberculosis (TB) continues to be a major health challenge with reported cases increasing worldwide. The World Health Organization reported an estimated global total of 10.6 million incident cases in 2022 [1]. Of these cases, 8,331 were reported within the United States which is a 5.9 % increase in cases from 2021 [2]. This resurgence of TB in nonendemic countries has long been attributed to factors such as migration and the HIV epidemic causing increased immunosuppression amongst patients [1,2].

Usually, severe cases of TB dissemination are seen in immunocompromised patients who can have extrapulmonary involvement in any organ system. In rare instances, this occurs in the bones and joints. Skeletal TB comprises 10 % of extrapulmonary TB cases with Extra Pulmonary Spinal TB (EPSTB) being the most common and the most dangerous [3,4]. Further spread to the sternum is rare accounting for only 0.3 % of cases [5,8].

The rarity alone; however, is not the only factor causing difficulty with diagnosis. EPSTB is well known to be a challenging diagnosis given its paucibacillary load in extrapulmonary specimens with microbiological and histological yield being reported to range between 42 % and 76 % [6,7]. Therefore, in cases without a definitive diagnosis and culture negative, as represented here in this case report, strong clinical suspicion must be correlated with diagnostic imaging and an extensive infectious work up. For our patient specifically, microscopic examination, culture, and percutaneous vertebral sampling were negative. The only positive infectious workup included a strong positive QuantiFERON-TB Gold result and highly suggestive MRI findings of atypical infection including tuberculosis.

Therefore, we present this case in order to share the importance of clinical evaluation and radiological imaging in a rare case of presumed spinal tuberculosis also involving the sternum in an immunocompetent United States born citizen with an indolent clinical course.

## Case report

**Fig. 1:**
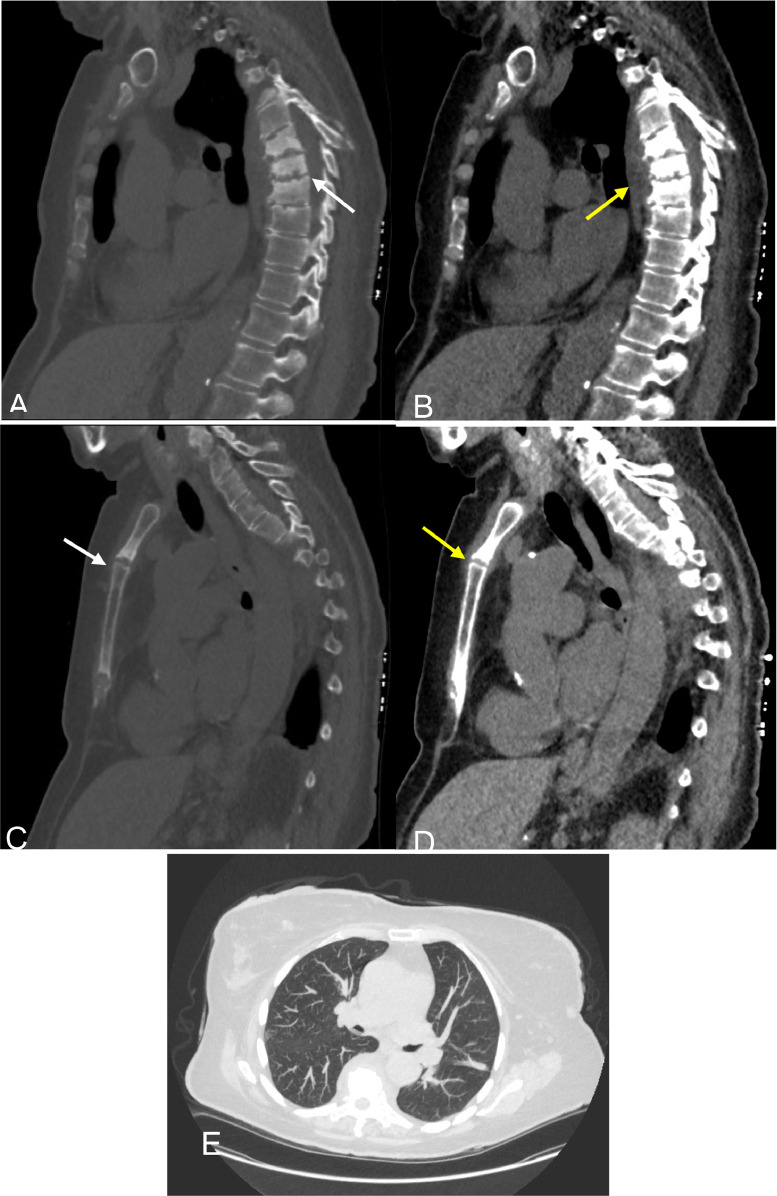
CT Chest: (A) new multilevel sclerotic vertebral lesions noted at T5-T8 with endplate irregularity (white arrow) (B) and associated paravertebral soft tissue phlegmon (yellow arrow). (C) Sclerosis (white arrow) and (D) soft tissue thickening (yellow arrow) also seen at the manubriosternal junction. (E) No pulmonary nodules, consolidation, or adenopathy demonstrated.

A 68 year old female with significant past medical history of breast cancer in remission and a 50 pack year smoking history presented for her annual low dose CT lung cancer screening exam. The intrathoracic CT findings were unremarkable without airspace consolidations, nodules, or adenopathy. However, incidental findings of erosive changes and multilevel sclerotic vertebral and sternomanubrial lesions were noted to be suspicious for spondylodiscitis and osteomyelitis (Fig. 1) respectively which prompted further workup.

Upon history and exam, the patient reported that she had been having progressively worsening back pain over the thoracic spine for 3 months. She denied fevers, chills, cough, shortness of breath, weight loss, and numbness and tingling of the extremities. All vitals were within normal limits and physical exam showed only midline tenderness to palpation of the thoracic spine and sternum without overlying erythema, induration, or fluctuance.

Labs showed a normal white count, low hemoglobin 10.2 g/dL, hematocrit at 31.6 %, elevated creatinine at 1.4 mg/dL, elevated calcium at 10.9 mg/dL, and elevated inflammatory markers with ESR at 85 mm/h and CRP at 11.34 mg/dL. Initial blood cultures were negative and total spine imaging was recommended.

**Fig. 2:**
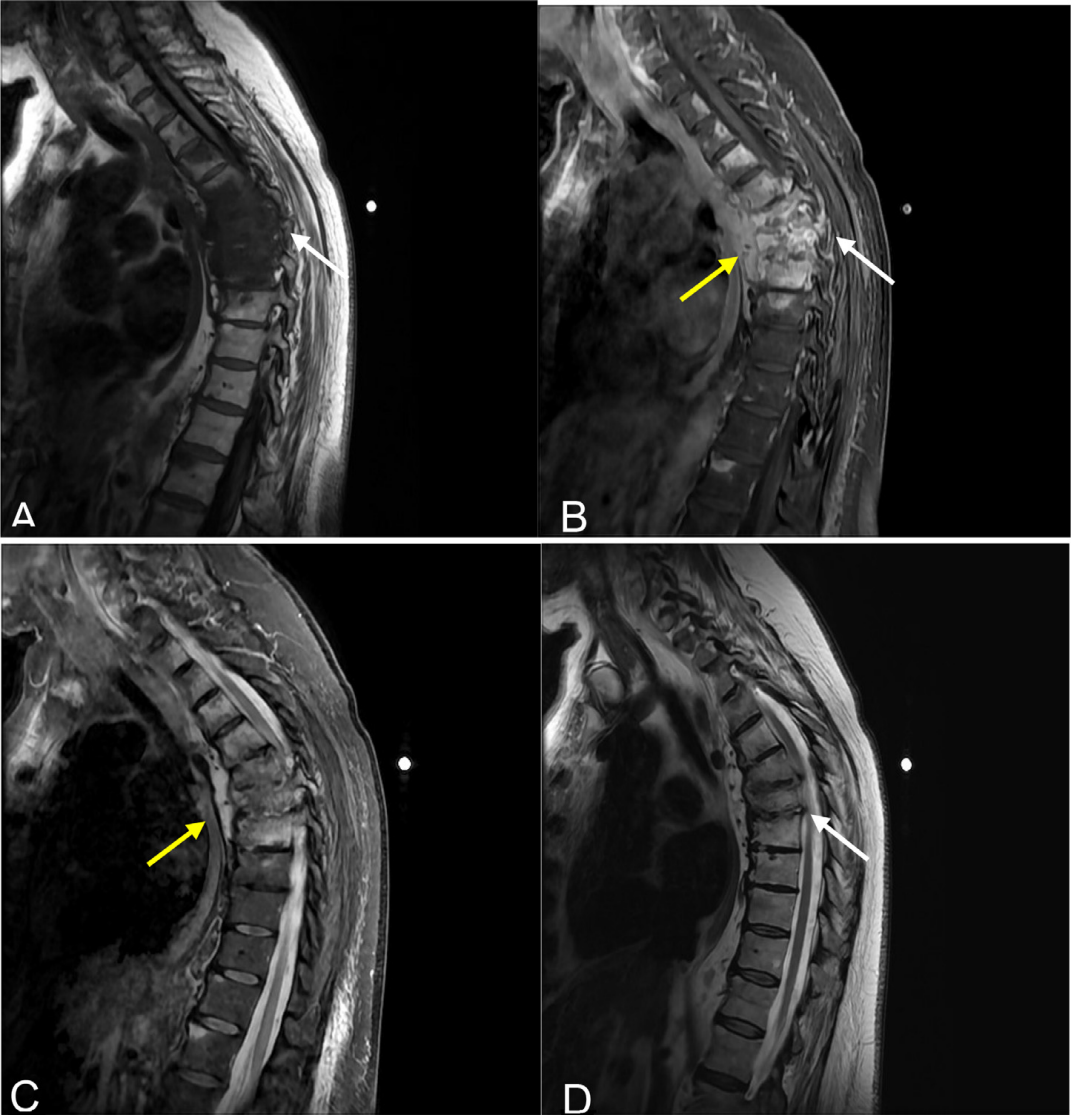
MRI thoracic spine sagittal: (A) T1 fluid attenuated inversion recovery (FLAIR), (B) postcontrast T1 with fat suppression, (C) STIR, and (D) T2. (A&B) Presence of extensive enhancing bone marrow changes at T5-T9 (white arrows) with (B&C) associated paravertebral phlegmon enhancement (yellow arrows). (D) Anterior wedge deformities of T6-T8 (white arrow). Additional skip lesions were noted in the cervical and lumbar spine (cervical involvement shown in Fig. 3).

**Fig. 3:**
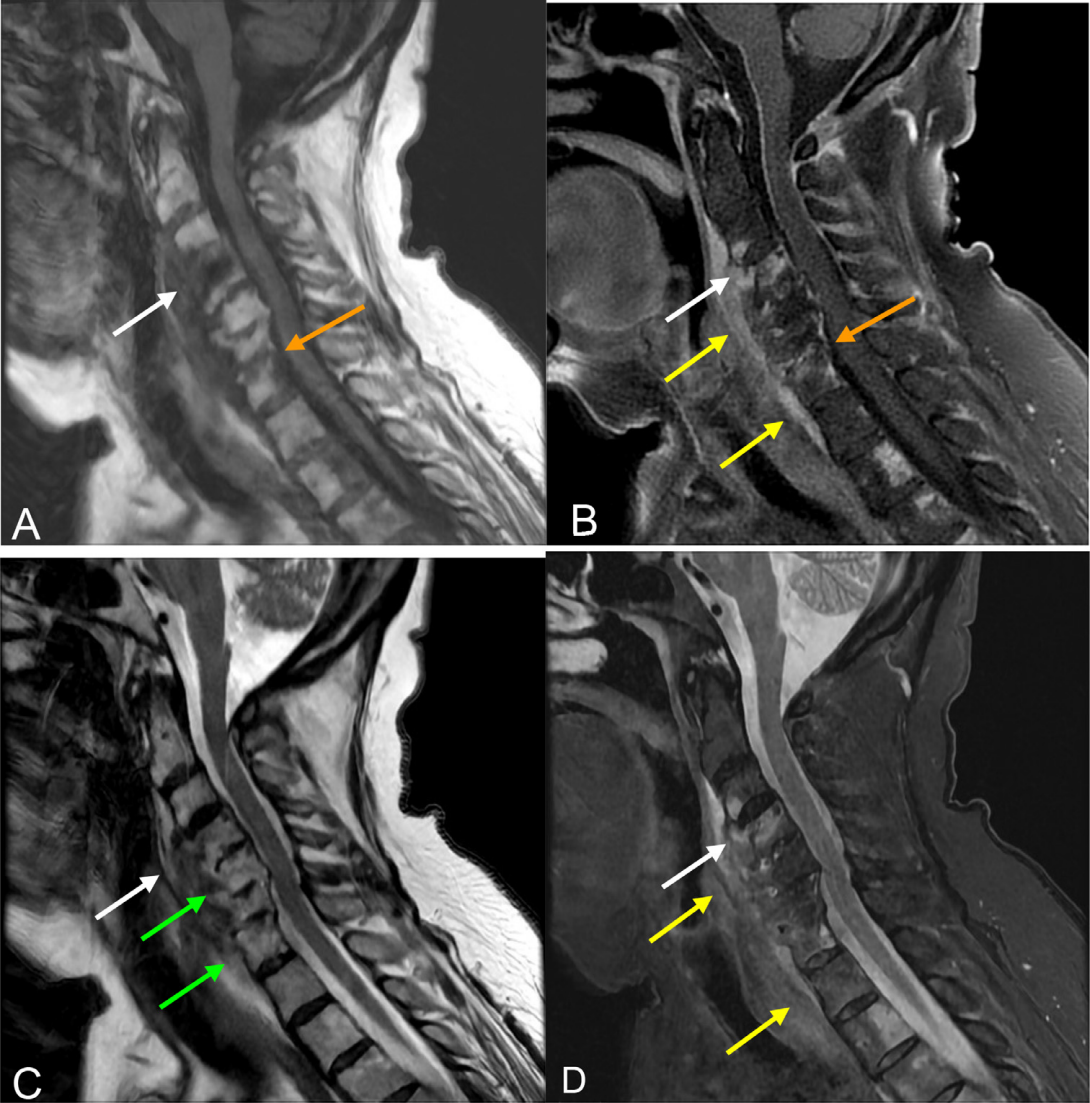
MRI cervical spine sagittal: (A) T1, (B) T1 fat-saturated (FS) post contrast, (C) T2, (D) STIR. (A-D) Additional skipped enhancing and destructive vertebral abnormalities were noted within the cervical spine primarily involving the endplates of C3-C4 (white arrow) and C6-C7 (orange arrow) (B&D) with relative sparing of T2 hypointense discs at the affected levels (green arrows). (D) Associated paravertebral edema which extends inferiorly along the anterior longitudinal ligament to the upper thoracic spine (yellow arrows).

**Fig. 4:**
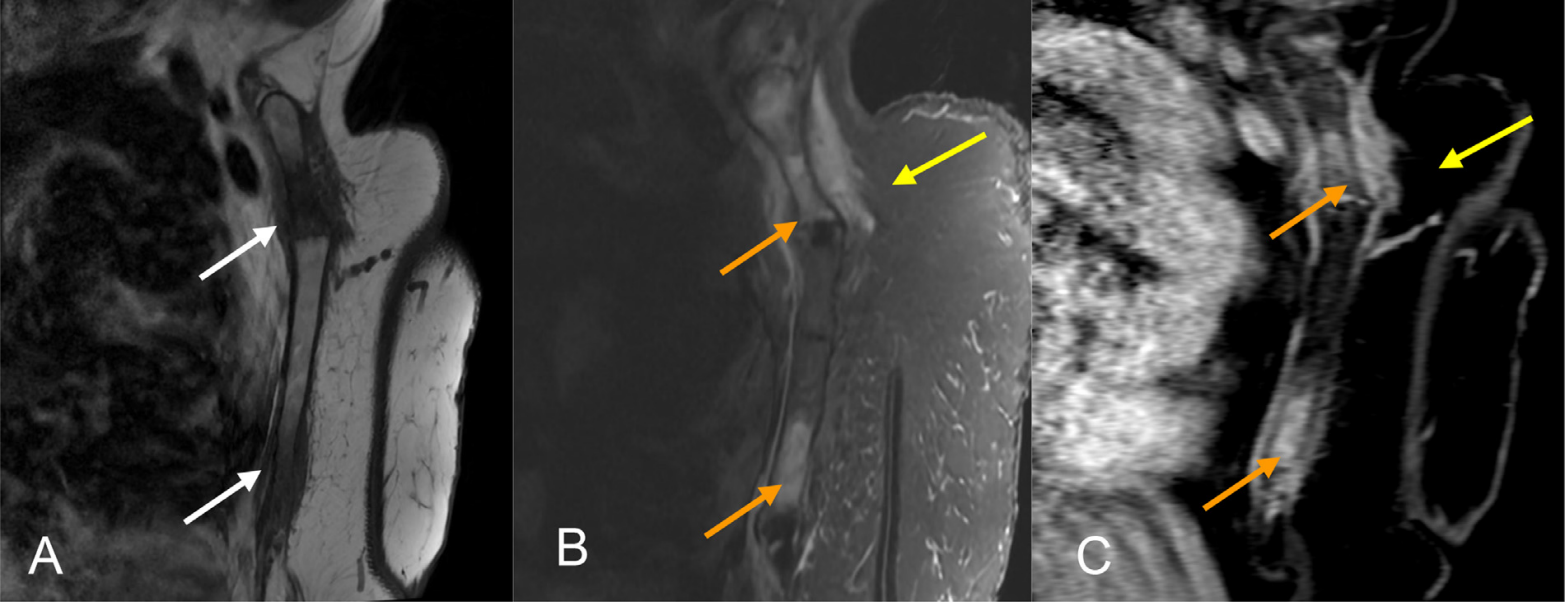
MRI chest sagittal: (A) T1, (B) STIR, (C) LAVA (T1 FS post contrast). (A) Abnormal T1 hypointense marrow replacement at the manubriosternal junction and lower sternum (white arrows) (B&C) with enhancing edema (orange arrows) and adjacent soft tissue inflammation (yellow arrows). No fluid collection or sinus tracts demonstrated.

**Fig. 5:**
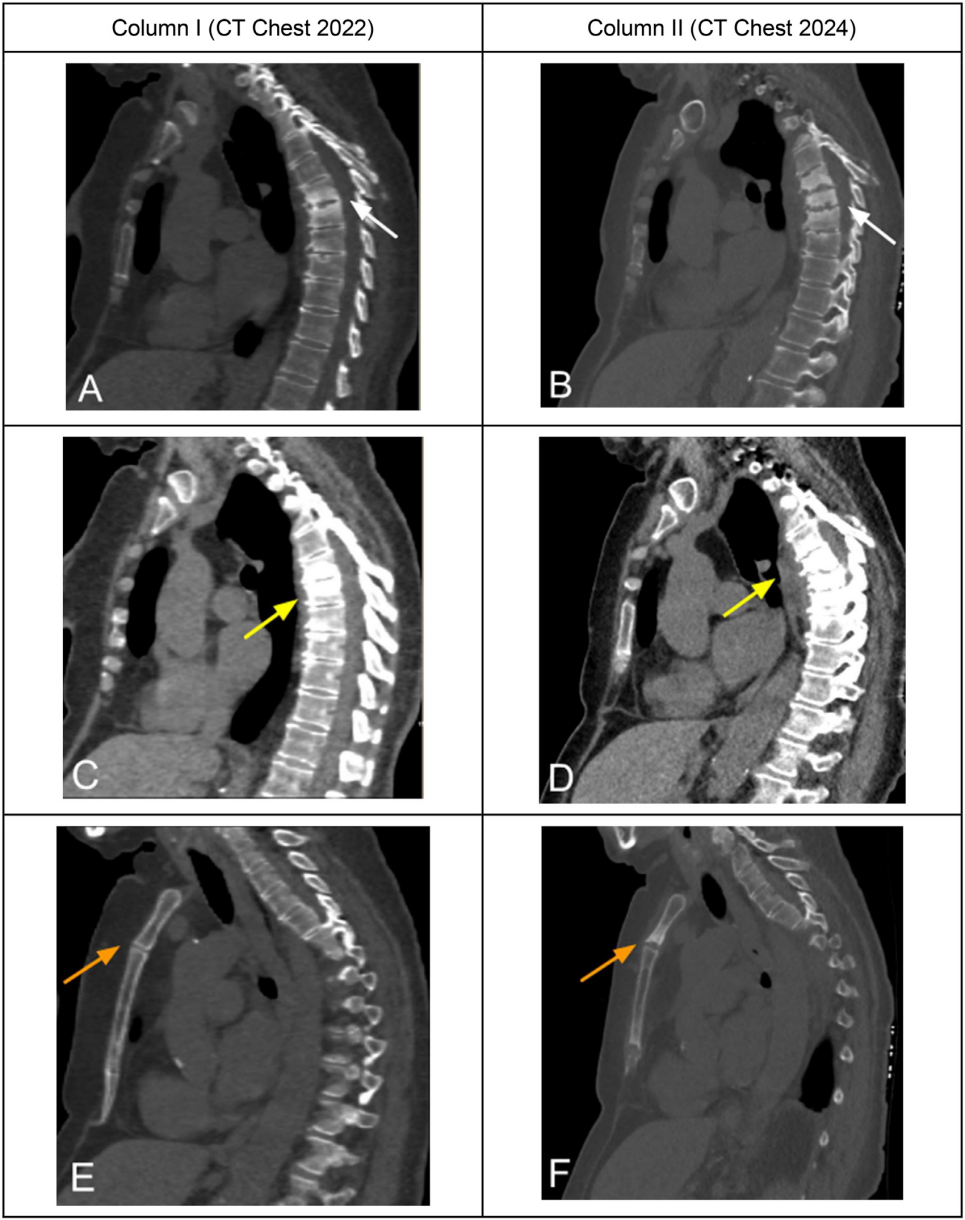
CT Chest 2022 in Column I and CT Chest 2024 in Column II showing progression of spread (A) CT Chest 2022 with bone window showing multilevel vertebral endplate sclerosis at T6-T7 (white arrows) (B) Shows interval increased vertebral sclerosis involving T5-T9 (white arrow). (C) Soft tissue window demonstrating thin paravertebral phlegmon (yellow arrow). (D) Interval increased paravertebral phlegmon (yellow arrow). (E) CT Chest 2022 has a lack of sternomanubrial findings (orange arrow) including sclerosis and soft tissue inflammation. (F) Shows interval development of sternomanubrial sclerosis (orange arrow) and soft tissue phlegmon (better visualized in Fig. 1).

**Fig. 6:**
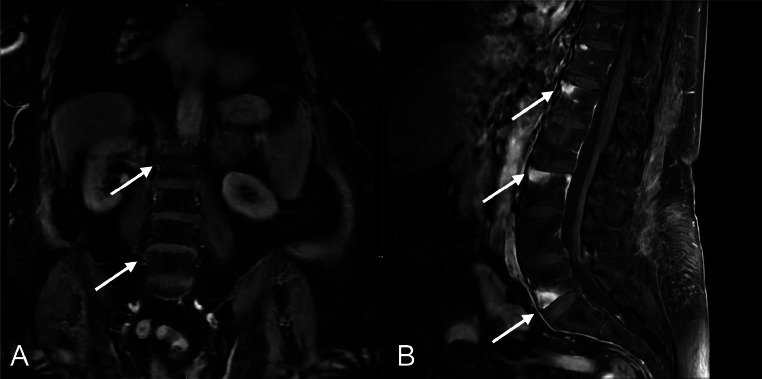
(A) MRI Abdomen 2023 T1 FS contrast enhanced sequence. L2-L5 vertebral bodies (white arrows) are visualized without end plate or soft tissue enhancement. No sagittal images available. (B) Compared to MRI L spine 2024 T1 fat saturated contrast enhanced sequence which shows marrow edema and vertebral endplate enhancement is seen at L1, L3, and L4-L5 (white arrows).

Contrast enhanced thoracic spine imaging demonstrated multilevel enhancing vertebral bone marrow edema with paravertebral soft tissue phlegm most prominent at levels T5-T9 consistent with osteomyelitis (Fig. 2). Similar noncontiguous abnormalities were also demonstrated throughout the spine in subsequent cervical and lumbar MRI studies predominantly involving the vertebral endplates with relative sparing of the discs suggestive of an atypical infection including tuberculosis (Fig. 3). Furthermore, retrospective imaging comparisons of previous low dose CT chest from 2022 (Fig. 5), 2 years prior, did not demonstrate sternomanubrial findings but did show irregular endplate sclerosis at T6-T7 with smaller paravertebral phlegmon suggesting general progression of multilevel spine and eventual sternomanubrial involvement (Fig. 4). MRI abdomen in 2023 showed lack of lumbar spine involvement (Fig. 6).

**Fig. 7:**
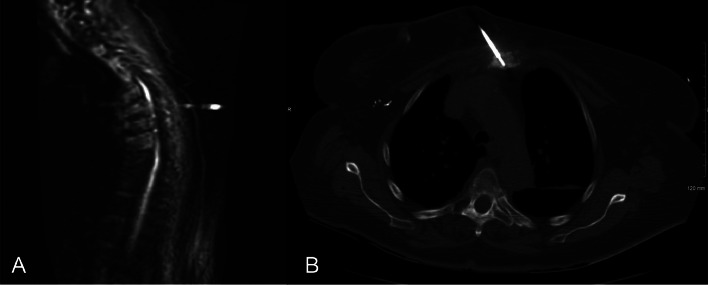
Biopsy images. (A) MRI bone biopsy showing location of sample retrieval at the T8 vertebral body. (B) Inferior manubrium CT bone biopsy of sclerosis.

Infectious workup included bone biopsies of the sternum and T8 vertebral body (Fig. 7). Bacterial gram stain and culture as well as examination for acid fast bacilli of both biopsy specimens returned negative. No sample was available for TB polymerase chain reaction (PCR) assay. Subsequent blood cultures, Karius analysis (lab tests detecting cell free DNA in various bacteria, DNA viruses, fungi, protozoa), brucella antibody screen, HIV antibody and antigen screen, and sputum TB cultures were also negative. Only pertinent positive finding was a strong QUANTIFERON TB Gold+ results with 2 antigen concentrations of 2.07 IU/mL and 1.75 IU/mL respectively (reference range of < 0.35 IU/mL). Further questioning regarding possible TB exposure was assessed, and the patient revealed that she worked as a coordinator for a children and family home in 2020 that required her to occasionally do in-home visits as well as visits within correctional facilities. She denied recent travel and exposure to known sick contacts. Therefore, due to the high suspicion of tuberculosis spondylitis, the patient was subsequently started on empiric antituberculous therapy including rifampin, isoniazid, ethambutol, pyrazinamide, and pyridoxine.

**Fig. 8:**
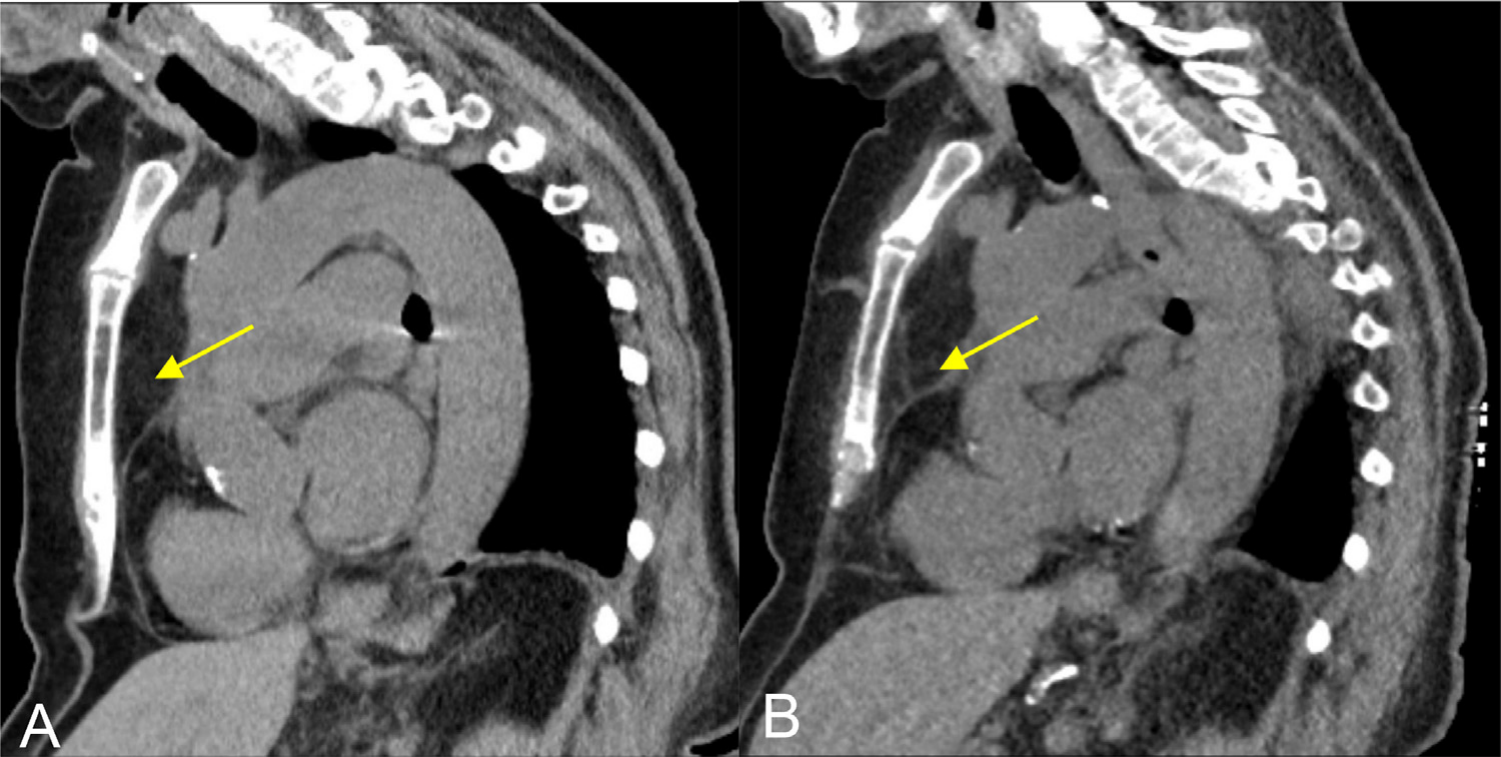
Most recent CT chest (left) compared to prior CT Chest 2 months prior (right). (A) Retrosternal soft tissue stranding (yellow arrow) showed improvement in the most recent follow-up study. T5-T9 endplate sclerosis was unchanged (not shown). (B) Prior CT Chest with retrosternal soft tissue inflammation (yellow arrow).

At her 1 month follow up, the patient showed clinical signs of improvement reporting a decrease in back pain while remaining on the same pain regimen. Her inflammatory markers, ESR and CRP, decreased from 85 mm/h to 20 mg/dL and from 11.34 mm/h to 0.76 mg/dL, respectively. Follow up imaging of the thoracic spine with CT showed unchanged sclerotic changes which is expected in a short time period although there was improvement in retrosternal inflammation (Fig. 8). Thus, she was continued on antituberculous therapy and has continued to show gradual clinical improvement with significant improvement of back pain at her 4 month follow up.

## Discussion

This case of presumed tuberculosis spondylitis is unique in that it presented itself on a low dose CT lung screening exam and showed not only multilevel spinal involvement, but also sternomanubrial spread which is extremely rare, especially in an immunocompetent United States born citizen with little to no symptoms or significant travel history.

Overall there are sparse reports of EPSTB spread to the sternum. TB of the sternum and clavicles accounts for only around 0.3 % of all skeletal TB cases even in endemic populations [5,8]. There are risk factors that increases one's likelihood of developing mycobacterial infections of the sternum such as recent sternotomy, BCG vaccination, and immunosuppression, notably human immunodeficiency virus; however, cases without a recognizable predisposing condition have been reported sporadically as well [9,10]. TB involvement of the sternum is not completely understood but is postulated to be either the result of reactivation of a latent focus of primary tuberculosis spreading hematogenously or through lymphatic dissemination or a direct extension from mediastinal lymph nodes [11].

EPSTB is commonly associated with spondylodiscitis or “Pott's disease”, particularly in the thoracolumbar junction most frequently, followed by the lumbar, cervical, and then sacral areas [12]. The spread EPSTB in our case could be inferred from the lack of findings on prior incidental imaging. Her thoracic spine was involved earlier with eventual involvement of the rest of the spine and sternum. Multiple vertebrae at one level are initially involved as the infection spreads via vascular anastomosis primarily affecting the anterior part of the vertebral body adjacent to the end plate [5,13]. However, skip lesions with sparing of the intervertebral discs occurs when surrounding ligaments become infected at which point subligamentous spread can occur both superiorly and inferiorly to other levels within the spine. As the infectious process continues, the structure of the vertebral bodies are compromised ultimately leading to collapse and anterior wedging of the vertebral body as well as extension into paravertebral soft tissues which was most prominent in our patient at thoracic levels T5-T9 [5,13]. These findings present on CT as areas of sclerosis and bony destruction with a corresponding paravertebral soft tissue enhancement on MRI as well as bone marrow changes that are hypointense on T1 and hyperintense on T2 weighted sequences [14]. Knowing the typical pattern and presentation of infectious spread by EPSTB can play a fundamental role in establishing diagnosis.

Clinical presentation of EPSTB is interesting in that constitutional symptoms are uncommon [10]. In our patient, specifically, there were no constitutional symptoms suggestive of underlying infection or pulmonary tuberculosis. Clinical presentation of sternal tuberculosis, however, are classically associated with pain, swelling, cold abscess, and sinus tract formation with various degrees of severity and involvement [8,12]. Our patient initially endorsed chest pain on palpation which subsequently resolved. No additional pertinent physical exam findings were present including swelling or sinus tract formation. We postulate that the patient's manubriosternal findings were subclinical and likely represented early findings given minimal destructive changes with increased enhancing bone marrow signal on MRI as seen in other case series [16].

Sternal and spinal image findings on the low dose CT lung cancer screening exam were incidental and started the imaging and infectious workup. However, microbiological testing can lead to conflicting results. EPSTB culture specimens have a maximum sensitivity rate of 80 % with culture positive specimens being reported as low as 33 % in large retrospective studies [6,7,15]. Therefore, QuantiFERON-TB Gold+ is often used as an adjunct in diagnosis since it has an 84 % sensitivity and 95 % specificity for prior or latent infection [12]. Although a positive or negative QuantiFERON-TB Gold+ result cannot rule in or exclude active TB infection respectively, it can be an useful adjunct in determining if there has been prior exposure or latent infection if there is a strong clinical concern for TB as was such in our case. This, along with radiological imaging of multilevel skipped vertebral lesions and subligamentous spread can suggest TB as the leading diagnosis. This was the case for our patient whose biopsy and culture results returned negative; however, her infectious workup including a strongly positive QuantiFERON-TB GOLD test and classic imaging findings initiated antituberculosis regiment including rifampin, isoniazid, ethambutol, pyrazinamide, and pyridoxine.

Upon clinical follow-ups 1 and 4 months later, patient reported singificant improvement of black pain. Multilevel vertebral sclerotic lesions and adjacent soft tissue thickening were unchanged on follow up CT imaging which may be expected in a short time period. However, retrosternal fat stranding decreased suggestive of improving inflammatory changes in addition to clinical improvement in the patient's back pain and decrease in inflammatory markers. This type of response to treatment is expected as radiological evidence of healing is known to lag behind clinical and laboratory results. Many patients may not exhibit any signs of improvement on radiographs or computed tomography scans for several months and for up to 3 months on MRI [12]. Therefore, it is important to not construe these imaging findings as signs of treatment failure. Poor treatment management is rather suspected with development of new lesions, worsening existing lesions, new bone damage or abscesses, or if no improvement is seen at repeat 6 month imaging [12]. In the interim, since clinical response precedes radiologic response, periodic clinical exams can be used to evaluate therapeutic effectiveness [17].

In conclusion, the diagnosis of EPSTB remains challenging due to an indolent clinical course and paucibacillary loads, especially in nonendemic areas. Sternal involvement is rare even in endemic settings although sternal tuberculosis should be considered if there is clinical suspicion for tuberculosis and suggestive radiographic findings including multilevel skipped vertebral lesions. MRI may be helpful in evaluating for marrow changes and soft tissue involvement which may detect early changes of TB.

## Patient consent

Written informed consent was obtained from the patient and they have given approval for their information to be published in this case report.
